# Yeast Monitoring of Wine Mixed or Sequential Fermentations Made by Native Strains from D.O. “Vinos de Madrid” Using Real-Time Quantitative PCR

**DOI:** 10.3389/fmicb.2017.02520

**Published:** 2017-12-20

**Authors:** Margarita García, Braulio Esteve-Zarzoso, Julia Crespo, Juan M. Cabellos, Teresa Arroyo

**Affiliations:** ^1^Instituto Madrileño de Investigación y Desarrollo Rural, Agrario y Alimentario, Madrid, Spain; ^2^Departament de Bioquímica i Biotecnologia, Facultat d’Enologia, Universitat Rovira i Virgili, Tarragona, Spain

**Keywords:** qPCR, native yeast, non-*Saccharomyces*, *Saccharomyces cerevisiae*, multi-starter fermentation, Malvar wine, sensorial analysis

## Abstract

There is an increasing trend toward understanding the impact of non-*Saccharomyces* yeasts on the winemaking process. Although *Saccharomyces cerevisiae* is the predominant species at the end of fermentation, it has been recognized that the presence of non-*Saccharomyces* species during alcoholic fermentation can produce an improvement in the quality and complexity of the final wines. A previous work was developed for selecting the best combinations between *S. cerevisiae* and five non-*Saccharomyces* (*Torulaspora delbrueckii, Schizosaccharomyces pombe, Candida stellata, Metschnikowia pulcherrima*, and *Lachancea thermotolorans*) native yeast strains from D.O. “Vinos de Madrid” at the laboratory scale. The best inoculation strategies between *S. cerevisiae* and non-*Saccharomyces* strains were chosen to analyze, by real-time quantitative PCR (qPCR) combined with the use of specific primers, the dynamics of inoculated populations throughout the fermentation process at the pilot scale using the Malvar white grape variety. The efficiency of the qPCR system was verified independently of the samples matrix, founding the inoculated yeast species throughout alcoholic fermentation. Finally, we can validate the positive effect of selected co-cultures in the Malvar wine quality, highlighting the sequential cultures of *T. delbrueckii* CLI 918/*S. cerevisiae* CLI 889 and *C. stellata* CLI 920/*S. cerevisiae* CLI 889 and, mixed and sequential cultures of *L. thermotolerans* 9-6C combined with *S. cerevisiae* CLI 889.

## Introduction

Alcoholic fermentation is a complex ecological and biochemical process where a succession of yeasts of several genera and species are able to convert must sugars into ethanol and carbon dioxide, as well as into important secondary metabolites (Barata et al., 2012; Sun et al., 2014; Albergaria and Arneborg, 2016). Even though *Saccharomyces* species are present at a low frequency on the surface of healthy grapes, *Saccharomyces cerevisiae* is considered the primary microorganism in the fermentation process and it is widely used in oenology (Martini et al., 1996; Fleet, 2003). However, during the last decade, non-*Saccharomyces* yeasts species have been proposed for winemaking as they could contribute to the improvement of wine quality (Ciani et al., 2014; Wang et al., 2015; Masneuf-Pomarede et al., 2016; Puertas et al., 2016). Thus, a new trend has emerged in winemaking using starter cultures composed by non-*Saccharomyces* yeasts, together with *S. cerevisiae* or for sequential fermentation with *S. cerevisiae*.

Molecular methods are showing useful results for detection and faster identification of microorganisms throughout the wine elaboration process (Ivey and Phister, 2011). Classical microbiological methods involving isolation coupled with the enumeration of microbes by plating can lead to misinterpretation of the real number of microorganisms since these methods fail to detect viable but non-culturable (VBNC) organisms (Divol and Lonvaud-Funel, 2005; Quirós et al., 2009; Salma et al., 2013; Wang et al., 2016) and minor populations present are difficult to detect on plates (Cocolin et al., 2013; David et al., 2014). Instead, molecular techniques, generally named culture-independent methods, are used for the identification of microorganism directly in the system through the study of their DNA or RNA without the need for isolation and cultivation, reducing detection time (Andorrà et al., 2008). Real-time quantitative PCR (qPCR) has been widely used in wine for microorganism detection during wine elaboration (Rawsthorne and Phister, 2006; Andorrà et al., 2008, 2010; Tofalo et al., 2012; Wang et al., 2014), providing significant advantages as the low detection level, the speed by which assays are performed, and the ability to quantify yeasts present following alcoholic fermentation.

In a previous work of García et al. (2017), small-scale fermentations were elaborated to study the oenological characterization of five non-*Saccharomyces* native yeast species under several co-culture conditions in combination with selected strain of *S. cerevisiae* CLI 889 to improve the organoleptic properties of the regional Malvar wines. There, the best inoculation process was selected depending on the non-*Saccharomyces* strain inoculated. Preferred sequential inoculations were elaborated with *S. cerevisiae* CLI 889 in combination with *Torulaspora delbrueckii* CLI 918 that produced wines with a higher fruity and floral aroma and lower ethanol content; with *Candida stellata* CLI 920 that increased the aroma complexity and glycerol content; and, with *Lachancea thermotolerans* 9-6C, produced an increase in acidity and floral and ripe fruit aroma. In the case of *Schizosaccharomyces pombe*, sequential fermentation was selected according to its fruity aroma score obtained after tasting. However, mixed cultures of *S. cerevisiae* with *Metschnikowia pulcherrima* CLI 457 and *L. thermotolerans* 9-6C was chosen due to a lower volatile acidity observed in final wines. Moreover, an increase of glycerol and ripe fruit aroma in the case of *M. pulcherrima* was observed, and for *L. thermotolerans* mixed culture the freshness, citric aroma, and full body were the main aspects to verify at the pilot scale.

Regarding these results, the aim of this work is to study yeast population evolution using real-time PCR during pilot winemaking trials under the best inoculation strategies. Moreover, validation of their positive effect on wine fermentation and wine quality was observed in the previous laboratory scale study (García et al., 2017) using sensory analysis.

## Materials and Methods

### Yeast Strains

The non-*Saccharomyces* strains used in this study are *T. delbrueckii* CLI 918, *S. pombe* CLI 1079, *C. stellata* CLI 920, *M. pulcherrima* CLI 457 and *L. thermotolerans* 9-6C, and *S. cerevisiae* CLI 889 strain were previously isolated on the Madrid winegrowing region and selected and characterized in our laboratories based on some established and desirable oenological criteria (Arroyo, 2000; Cordero-Bueso et al., 2013, 2016).

### Wine Fermentation and Sampling

The pilot winemaking (stainless steel tanks with 16 L of must) was performed at IMIDRA’s experimental cellar is located in the Madrid winegrowing region, Spain (40°31′ N, 3°17′ W and 610 m altitude). Grapes were collected from Malvar (*Vitis vinifera* cv.) white grape variety to elaborate the wines, which were obtained in accordance with the cellar standard practices for harvest. Musts were racked, homogenized, and dislodged statically at 4°C to clarify and be sulfited (50 ppm). Musts obtained from two different vineyards, Must I and Must II, showed 1095 and 1099 g L^-1^ of density, pH values were 3.05 and 3.15, titratable acidity (expressed as g L^-1^ of tartaric acid) was 5.7 and 4.8, and yeast assimilable nitrogen (YAN) values were 218 and 100 mgN L^-1^, respectively.

Triplicate fermentations were carried out in stainless steel tanks with 16 L of fresh Malvar must at a controlled temperature of 18°C without agitation and, the tanks were locked to maintain anaerobiosis throughout alcoholic fermentation (CO_2_ was released through a sterile Müller valve with 96% H_2_SO_4_). Tanks were inoculated with a *pied de cuve* until a concentration of 10^6^ cells mL^-1^ of each yeast strain. These inocula were achieved by an overnight culture of the different yeast strains in sterile must of the same variety prepared away from the cellar. Preselected combinations between *S. cerevisiae* CLI 889 and the different non-*Saccharomyces* species were the best results in García et al. (2017). We named mixed fermentation when both strains are inoculated at the same time, and in sequential fermentation, the non-*Saccharomyces* culture was inoculated at first and the addition of *S. cerevisiae* takes place when the wine contains 5% alcohol (*v*/*v*). The trials tested in must I, were: sequential culture of *T. delbrueckii* CLI 918 and *S. cerevisiae* CLI 889 strains (s-Td/ScI); mixed culture of *M. pulcherrima* CLI 457 and *S. cerevisiae* CLI 889 strains (m-Mp/ScI); and pure culture of *S. cerevisiae* CLI 889 (p-ScI), culture considered as control. The combinations in Malvar must II were: sequential culture of *S. pombe* CLI 1079 and *S. cerevisiae* CLI 889 (s-Sp/ScII); sequential culture of *C. stellata* CLI 920 and *S. cerevisiae* CLI 889 (s-Cs/ScII); mixed culture of *L. thermotolerans* 9-6C and *S. cerevisiae* CLI 889 (m-Lt/ScII); sequential culture of *L. thermotolerans* 9-6C and *S. cerevisiae* CLI 889 (s-Lt/ScII); and pure culture of *S. cerevisiae* CLI 889 (p-ScII) as a control.

The fermentation process was monitored daily though density, °Baumé, and temperature measurements until constant density (lower than 1000 g L^-1^). Samples were taken for every tank during the vinification process. Samples (1 mL) for qPCR analyses were centrifuged and pellets were immediately cryo-preserved. For total yeast counts, samples were spread on yeast extract peptone dextrose (YPD) plates and on lysine agar medium [0.25% L-Lysine monohydrochloride (Sigma–Aldrich, St. Louis, MO, United States), 1.17% yeast carbon base (Difco, Detroit, MI, United States), and 2% agar, w/v], a selective medium for the differentiation of non-*Saccharomyces* yeast populations which does not support the growth of *S. cerevisiae* (Walters and Thiselton, 1953). One week after fermentation finished, the wines were bottled after racking and adding 50 ppm SO_2_.

### Oligonucleotides

Specific-species primers were designed in this work from conserved sequences of the variable D1/D2 domains of the 26S rDNA gene. Generated sequences were aligned with sequences of strains of the same species (**Table 1**) available at the National Centre for Biotechnology Information (NCBI)^1^ using Clustal W multiple-sequence alignment (Thompson et al., 1994). The primer design was performed using the Primer3Plus program^2^. Furthermore, the properties of each primer were verified by NIST Primer Tools^3^. Primers used in this study (**Table 2**) were synthetized by TIB MOLBIOL (Berlin, Germany). Moreover, conventional and real-time PCR were carried out using a range of yeast species to verify the specificity of each primer set.

**Table 1:** List of the accession numbers from GenBank of the sequences used for primer design.

Yeast species	Strain/isolate number	Accession number	Primer^b^					
			SC	Tods	SP1	CS1	MP2	LTH2
*Saccharomyces cerevisiae*	CLI 889^a^	MF001376	+	–	–	–	–	–
	GS1-3	FJ912839						
	N9323	EU268657						
	cs56	JX129910						
	CBS 2811	KY109393						
*Torulaspora delbrueckii*	CLI 918^a^	JQ707782	–	+	–	–	–	–
	t15-CTR-7	HQ845012						
	BBMV3FA5	KF735113						
*Schizosaccharomyces pombe*	CLI 1079^a^	MF001377	–	–	+	–	–	–
	CLI 1085	JQ804983						
	ATCC 16979	KF278469						
	NCYC 3748	JF951752						
*Candida stellata*	CLI 920^a^	JQ707776	–	–	–	+	–	–
	CBS 2843	EF452199						
	NX8A	EF564405						
*Metschnikowia pulcherrima*	CLI 457^a^	MF001378	–	–	–	–	+	–
	cs51	JX129913						
	N213	EU268661						
*Lachancea thermotolerans*	9-6C^a^	MF001379	–	–	–	–	–	+
	CLI 1219	JQ707778						
	cs240	JX129903						
*Pickia kudriavzevii*	CLI 1216	JQ707777						
	cs280	JX129897						
	cs336	JX129895						
*Candida zemplinina*	cs271	JX129898						
*Candida apicola*	cs15	JX129912						
*Hanseniaspora uvarum*	cs247	JX129900						
	B-1-7	FJ842088						
*Hanseniaspora guilliermondii*	A11-1-5	EU386752						
	CEC 13A2	KR069091						
*Issatchenkia terricola*	cs212	JX129906						
*Zygosaccharomyces bailii*	N2314	EU268642						
*Zygosaccharomyces rouxii*	CECT 10425	KX539237						

*Species and strain designations were included. ^a^These sequences belong to the yeast strains used in this work. ^b^Results by conventional PCR with primers used in this work (+, presence of PCR product; -, absence of PCR product).*

**Table 2:** Primer sequences used for real-time quantitative PCR analysis.

Yeast species	Primer name	Sequence 5′-3′	Reference
*Saccharomyces cerevisiae*	SC1	GAAAACTCCACAGTGTGTTG	Zott et al., 2010
	SC2	GCTTAAGTGCGCGGTCTTG	
*Torulaspora delbrueckii*	Tods L2	CAAAGTCATCCAAGCCAGC	Zott et al., 2010
	Tods R2	TTCTCAAACAATCATGTTTGGTAG	
*Schizosaccharomyces pombe*	SP1-F	AGTGAAGCGGGAAAAGCTCA	This work
	SP1-R	ATCGACCAAAGACGGGGTTC	
*Candida stellata*	CS1-F	AGTAACGGCGAGTGAACAGG	This work
	CS1-R	GGCTATCACCCTCTATGGCG	
*Metschnikowia pulcherrima*	MP2-F	AGACACTTAACTGGGCCAGC	This work
	MP2-R	GGGGTGGTGTGGAAGTAAGG	
*Lachancea thermotolerans*	LTH2-F	CGCTCCTTGTGGGTGGGGAT	This work
	LTH2-R	CTGGGCTATAACGCTTCTCC	

*The microorganisms’ targets for each couple of primers are included.*

### DNA Extraction and Real-Time PCR Assays

Yeast cell pellets were washed with sterile distilled water, and the pellets were resuspended in 700 μL of AP1 buffer (DNeasy Plant Mini Kit; QIAGEN, Valencia, CA, United States) and transferred in a 2-mL microcentrifuge tube containing 1 g of 0.5 mm-diameter glass beads. The tubes were shaken in a mixer mill (Retsch GmbH, Haan, Germany) for 3 min at the maximum rate and then centrifuged at 10,000 rpm for 1 min. Then, the supernatant was transferred to a sterile tube and purified using DNeasy Plant Mini Kit (QIAGEN) according to the manufacturer’s instructions.

qPCR was performed on an Applied Biosystems Prism 7500 sequence detection system (Applied Biosystems, Carlsbad, CA, United States). PCR amplification was conducted in optical-grade 96-well plates (Applied Biosystems) and each 25 μL reaction mixture containing 5 μL of DNA, 0.7 μM of each respective primer, and 12.5 μL of SYBR Green Master Mix (Roche Diagnostics GmbH, Mannheim, Germany). Each reaction was made in triplicate. The reaction conditions were an initial step at 95°C for 10 min and 40 cycles of 95°C for 15 s, 60°C for 1 min and 72°C for 30 s. The C_T_ was determined automatically by the instrument. The coefficients of efficiency (E) were calculated using the formula E = (10^-1/slope^) – 1 (Higuchi et al., 1993).

### Standard Curves

Standard curves for each yeast strain were created by plotting the cycle threshold (C_T_) values of the qPCR performed with dilution series of yeast cells (10^7^ to 10^3^ cells mL^-1^) against the log input cell mL^-1^ (ABI PRISM 7500 sequence detection system, Applied Biosystems). Standard curves were created for the six yeast strains used in this work.

### Artificial Contamination of Wines

Commercial Tempranillo red wine and Malvar white wine, previously sterilized by filtration, and YPD liquid medium were artificially contaminated with *T. delbrueckii* CLI 918, at known concentrations (10^6^ to 10^2^ cells mL^-1^). After incubation of 24 h at 20°C, DNA was isolated as indicated before for qPCR analysis. Standard curves for quantification of samples and determination of amplification efficiency were constructed. These dilutions were also plated on YPD agar and incubated 1 week at 28°C to obtain the number of CFU per milliliter using an easySpiral^®^ plater (Interscience, St. Nom, France).

### Study of *Saccharomyces cerevisiae* at the Strain Level

Microsatellite multiplex PCR analysis was used to check the presence of *S. cerevisiae* CLI 889 in the different types of elaboration, using the highly polymorphic loci SC8132X, YOR267C, and SCPTSY7 (Vaudano and Garcia-Moruno, 2008). The analysis was performed according to Cordero-Bueso et al. (2011) and Tello et al. (2012).

### Analytical Determination

Oenological parameters as alcohol degree, pH, volatile acidity, total acidity, reducing sugars, glycerol, malic acid, and lactic acid were measured by Fourier transform infrared spectroscopy in the laboratories of Liec Agroalimentaria S.L. (Manzanares, Spain). An accredited laboratory for physico-chemical analysis in wines to conform to UNE-EN ISO/IEC 17025:2005 rules. YAN was determined in must by the formol titration method (Gump et al., 2002).

Quantification of major volatile compounds was carried out in a GC Agilent 6850 with a FID detector equipped with a column DB-Wax (60 m × 0.32 mm × 0.5 μm film thickness) from J&W Scientific (Folsom, CA, United States). Analyses were done according to Gil et al. (2006) and Balboa-Lagunero et al. (2013).

### Sensorial Analysis

The final wines were subjected to two sensory analyses, triangle tests (ISO 4120:2007) and descriptive analysis by a trained panel of seven skilled judges from the IMIDRA Institute. Using triangle tests, the judges determine if a sensory difference exists between the wines tested. Sensory descriptive analysis was based on the description of attributes of the wines though 15 aroma and taste descriptors, and the panelists were asked about their preferences. These attributes were estimated on basis a scale from 1 (low intensity) to 10 (high intensity) and total scores were obtained as the mean and standard deviation of seven evaluations (Arroyo et al., 2009; Balboa-Lagunero et al., 2013).

### Statistical Analysis

Analysis of variance was carried out by an ANOVA Tukey test to determine significant differences (α = 0.05) between the samples with their respective fermentation control. PCA analysis was performed to identify the most influential oenological parameters and volatile compounds in the different types of cultures. The data were analyzed with SPSS Statistics 21.0 Software for Windows (SPSS, Inc., Chicago, IL, United States).

## Results

### Primer Design, Specificity and Sensitivity of qPCR

Primers proposed in this work were designed on the variable D1/D2 domains of 26S rDNA gene, amplifying products between 100 and 150 bp in length. Primers for the quantification of *T. delbrueckii* and *S. cerevisiae* strains were designed by Zott et al. (2010) from the region of internal transcribed spacers (ITSs) of the ribosomal DNA region. The other primers used were designed for this work according to those described in the material and methods sections. Sequences for all primers are listed on the **Table 2**.

Each pair of primers exhibited *in silico* specific homology to only species for which were designed. Additionally, conventional PCR was performed using purified DNA from the yeast species used in this study and different strains belonging to the yeasts species *Candida vini, Wickerhamomyces anomalus, Zygosaccharomyces bailii, Meyerozyma guilliermondii, Pichia membranifaciens, Priceomyces carsonii*, and *Lachancea fermentati*, the most usual species isolated during spontaneous fermentation of Malvar must in the experimental cellar of IMIDRA (Cordero-Bueso et al., 2013), which are also included in the IMIDRA Institute Collection. Amplifications were observed only for those species which the primers that were specifically developed (**Table 1**).

To determine the standard curves qPCR, YPD cultures of each strain containing 10^7^ cells mL^-1^ were serially diluted 10-fold until 10^3^ cells mL^-1^ and DNA were extracted from 1 ml of each dilution. The DNA was then amplified by qPCR and standard curves were constructed. The slope, intersection, correlation coefficient (R^2^), and efficiency of the standard curves obtained are shown in **Table 3**. The assays were linear over five orders of magnitude and, the detection limit for all yeast species was 10^3^ cells mL^-1^.

**Table 3:** Standard curves performed for each yeast species.

Yeast	Slope	Intersection	*R*^2^	Efficiency (%)
*S. cerevisiae*	–3.17 ± 0.04	37.20 ± 0.26	0.997 ± 0.00	106.7 ± 1.97
*T. delbrueckii*	–3.27 ± 0.13	38.15 ± 0.61	0.996 ± 0.00	102.2 ± 5.62
*S. pombe*	–3.12 ± 0.05	37.58 ± 0.45	0.999 ± 0.00	108.9 ± 1.06
*C. stellata*	–3.19 ± 0.21	37.53 ± 0.81	0.998 ± 0.00	105.9 ± 8.72
*M. pulcherrima*	–3.29 ± 0.01	39.06 ± 0.04	0.992 ± 0.00	101.3 ± 0.39
*L. thermotolerans*	–3.11 ± 0.18	37.97 ± 0.41	0.993 ± 0.00	109.4 ± 3.29

*The slope, intersection, correlation coefficient (R^*2*^), and efficiency of standard curves of *S. cerevisiae, T. delbrueckii, S. pombe, C. stellata, M. pulcherrima*, and *L. thermotolerans* were determined by qPCR analysis. Mean ± standard deviation of triplicate qPCR amplifications are shown. Efficiency was estimated by the formula E = (10^-*1*/*slope*^) – 1.*

### Quantification in Artificially Contaminated Wines

**FIGURE 1:**
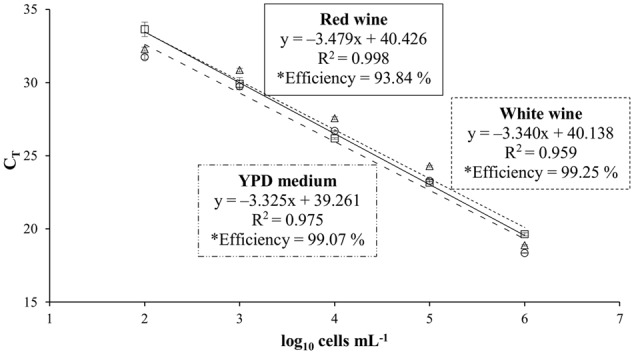
Standard curves obtained for *Torulaspora delbrueckii* CLI 918 from YPD culture (○, –..–), white wine (∆, - - - -), and red wine (□, —). C_T_ values of standard curves from YPD medium, white wine and red wine are the averages of three individual repetitions. ^∗^Efficiency was estimated by the formula E = (10^-1/slope^) – 1.

To study the influence of the wine matrix on the efficiency of the real-time PCR system, standard curves using artificial contaminated wines with *T. delbrueckii* CLI 918 strain were obtained from white (Malvar) and red (Tempranillo) wines, and YPD (control) cultures (**Figure 1**). *T. delbrueckii* CLI 918 strain was used to study this influence. Detection limits for all curves were 10^2^ cells mL^-1^ being linear over five orders of magnitude. The correlation coefficients, slopes, and efficiencies of the amplification of standard curves are shown in **Figure 1**. It could be possible to observe that the efficiency of qPCR in red wine is lower than white wine and YPD medium, however the differences observed were not statistically significant (*p* < 0.05). This type of analysis was also done for other yeast species used in this study (data not shown) and the results agreed with the *T. delbrueckii* trial.

### Yeast Inoculated Population Analysis by qPCR during Alcoholic Fermentation

**FIGURE 2:**
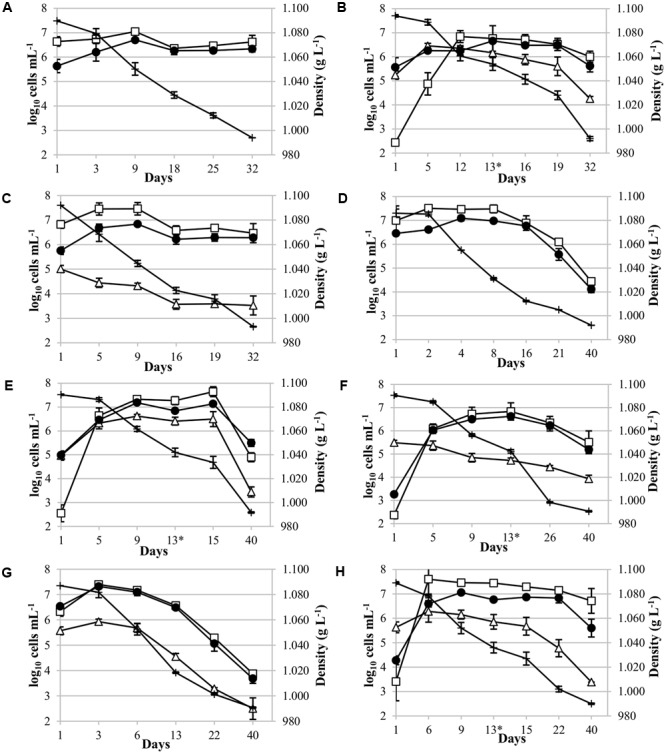
Yeast population dynamics during Malvar fermentation. Results, expressed as log_10_ cells mL^-1^, were obtained using YPD culture media (●) and qPCR analysis [(□) for *Saccharomyces cerevisiae*; (∆) for non-*Saccharomyces* strains] and, density values (+) expressed in g L^-1^. Species population analyzed: **(A)** Pure culture of *S. cerevisiae* CLI 889, control p-ScI; **(B)** sequential culture of *T. delbrueckii* CLI 918 and *S. cerevisiae* CLI 889 (s-Td/ScI); **(C)** mixed culture of *Metschnikowia pulcherrima* CLI 457 and *S. cerevisiae* CLI 889 (m-Mp/ScI); **(D)** pure culture of *S. cerevisiae*, control p-ScII; **(E)** sequential culture of *Schizosaccharomyces pombe* CLI 1079 and *S. cerevisiae* CLI 889 (s-Sp/ScII); **(F)** sequential culture of *Candida stellata* CLI 920 and *S. cerevisiae* CLI 889 (s-Cs/ScII); **(G)** mixed culture of *Lachancea thermotolerans* 9-6C and *S. cerevisiae* CLI 889 (m-Lt/ScII); and **(H)** sequential culture of *L. thermotolerans* 9-6C and *S. cerevisiae* CLI 889 (s-Lt/ScII). Asterisk in graphics indicates the day of inoculation of *S. cerevisiae* strain in sequential cultures.

qPCR analysis was used to analyze the dynamics of five non-*Saccharomyces* yeasts inoculated, revealing that they were present throughout the alcoholic fermentation. A culture-dependent technique on YPD plates were used to follow the evolution of total cultivable yeasts (**Figure 2**).

Pure cultures of *S. cerevisiae* CLI 889 (p-ScI and p-ScII) used as controls in the fermentations with must I and must II presented different population dynamics. The control p-ScI slowly started to ferment, achieving the highest population at day 9, its fermentation finished with a population of 2.5 × 10^6^ cells mL^-1^ after 32 days (**Figure 2A**). Instead, p-ScII culture reached the greatest population on the second day of fermentation, finishing with 2.7 × 10^4^ cells mL^-1^ after 40 days (**Figure 2D**). The amount of sugar daily transformed in these pure cultures when 50% of the sugar content had been consumed (V_50_) was higher in p-ScII (V_50_: 16.23) than p-ScI (V_50_: 13.30); finally, the p-ScI culture ended the fermentation with 9.86 g L^-1^ of reducing sugars and 13.5% (*v*/*v*) of ethanol, while p-ScII was able to consume the sugars present in the grape must and finished with 13.0% (*v*/*v*) of ethanol (Supplementary Table S1).

Regarding to mixed cultures (**Figure 2C** for *M. pulcherrima* and 2G for *L. thermotolerans*), on **Figure 2C** it could be possible to observe a small increase of *S. cerevisiae* population until day 9, after that a decrease and a maintenance in its population were observed. In contrast, *M. pulcherrima* population decreased from the beginning of the fermentation, finishing with three orders of magnitude lower than its control (p-ScI) at the end of fermentation after 32 days of vinification. The density values decreased to day 16, when the slow decrease of density coincided in time with the population stabilization of *M. pulcherrima* and *S. cerevisiae*. In the case of *L. thermotolerans* mixed fermentation (**Figure 2G**), there was an increase of this yeast population at the beginning, and after 6 days, a decrease was observed. In the whole fermentation process, the *S. cerevisiae* population was higher than *L. thermotolerans* population. The growth profile of *S. cerevisiae* in this mixed culture (**Figure 2G**) shows a high similarity with its control p-ScII (**Figure 2D**). Both cases on mixed fermentations, the fermentation takes the same time to reduce the density than the controls, and the residual sugars in final wines were also similar to their respective controls.

For sequential cultures, *S. cerevisiae* CLI 889 strain was inoculated at day 13 (represented by the asterisk in the graphics). It is worth noting that the native *S. cerevisiae* population increased between four and five orders of magnitude during the beginning of sequential fermentations, however, an improvement of the fermentation rate has been observed after *S. cerevisiae* inoculation (**Figures 2B,E,F,H**). After microsatellites multiplex PCR analysis to check the presence of *S. cerevisiae* CLI 889 strain from its day of inoculation (day 13) over another *S. cerevisiae* presented in the cellar environment, we found that the microsatellite pattern of the strain inoculated was exhibited by all the isolates analyzed. In **Figure 2B** it is possible to observe that the highest concentration of *T. delbrueckii* CLI 918 was achieved after 5 days, remaining at this level during the alcoholic fermentation, and finishing with the greatest final concentration in comparison with the other non-*Saccharomyces* tested in the sequential cultures. Although this fermentation takes the same length that its control, they need 32 days to reduce the density to lower than 1000 g L^-1^, the amount of residual sugars is different, showing lower concentrations for the sequential inoculation than its control (Supplementary Table S1). In the *S. pombe*/*S. cerevisiae* sequential culture (**Figure 2E**), an increment of *S. cerevisiae* population after *S. cerevisiae* CLI 889 inoculation can be observed. The *S. pombe* CLI 1079 population is maintained high during the fermentation even after *S. cerevisiae* is added. At the end of vinification, this non-*Saccharomyces* strain finished with approximately one order of magnitude less than *S. cerevisiae* population. *C. stellata* CLI 920 which seemed to be less competitive in this type of inoculation, presented a number of cells two orders of magnitude lower than *S. cerevisiae* from the day 9 (**Figure 2F**). This strain in sequential fermentation (**Figure 2F**) presented its higher counts after the first 24 h, then started to decrease until the end of fermentation (day 40). In this case, however, the inoculation of the *S. cerevisiae* strain produces an improvement of the fermentation rate, showing on **Figure 2F** a high reduction on the density, but the amount of *S. cerevisiae* was not changed. *L. thermotolerans* in sequential culture (**Figure 2H**) remained at high and relatively stable cell levels until day 15 when its population decreased more quickly, ending with three orders of magnitude less than *S. cerevisiae* at the end of fermentation, probably due to *S. cerevisiae* CLI 889 inoculation at day 13, which also produced a decrease of density.

### Analytical Determination of Wines

The main oenological parameters analyzed are listed in Supplementary Table S1, which shows that sequential fermentations produced wines so different to their control. Most of the cases the differences involve three or more parameters, while on mixed fermentations the differences with respect to the controls are reduced to a few parameters. Although the differences observed in the ethanol produced among the different fermentations exhibited a significant difference, these differences are lower than 0.5% (*v*/*v*), having no consideration for establish differences due to this parameter. However, the differences with respect to the control can be observed using other parameters, such as glycerol or malic acid. Volatile compounds analyzed (Supplementary Table S2) do not show significant differences on single compounds, but they have been observed when clusters of compounds have been conducted.

**FIGURE 3:**
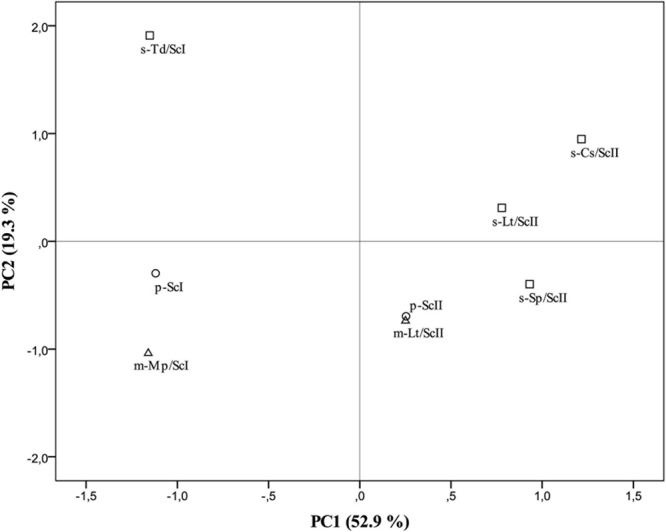
Results of the principal component analysis (PCA) performed on the oenological parameters and volatile compound data. Mean of triplicates wine samples derived from pure cultures of *S. cerevisiae* (p-ScI and p-ScII), sequential culture of *T. delbrueckii* and *S. cerevisiae* (s-Td/ScI), mixed culture of *M. pulcherrima* and *S. cerevisiae* (m-Mp/ScI), sequential culture of *S. pombe* and *S. cerevisiae* (s-Sp/ScII), sequential culture of *C. stellata* and *S. cerevisiae* (s-Cs/ScII), mixed (m-Lt/ScII) and sequential (s-Lt/ScII) cultures of *L. thermotolerans* and *S. cerevisiae* in the plane formed by the two first principal components. Pure cultures represented by circles (○), mixed cultures by triangles (∆), and sequential cultures by squares (□).

To confirm the differences among pure cultures of *S. cerevisiae* (p-ScI and p-ScII, considered as controls) and co-culture-fermented wines, a principal component analysis (PCA) was elaborated (**Figure 3**) from all data obtained from the analysis of oenological parameters and volatile compounds (Supplementary Tables S1, S2). The first two principal components, PC1 and PC2 accounted for 72.23% of total variance (**Figure 3**). PC2, which is mostly formed by volatile compounds (the impact of each parameter on the component is indicated in brackets) as ethyl isovalerate (0.971), ethyl-3-hydroxybutyrate (0.949), 1-butanol (0.914), isoamyl acetate (0.750), ethyl butyrate (0.614), and ethyl hexanoate (0.606), allowed us to differentiate the different types of culture with non-*Saccharomyces* species in combination with the *S. cerevisiae* strain, while the main parameters for PC1 were hexanoic acid (0.989), octanoic acid (0.982), 1-hexanol (0.979), isovaleric acid (0.965), diacetyle (0.961), isoamyl alcohol (0.929), β-phenylethyl alcohol (0.927), isobutanol (0.901), and pH (0.876), differentiating the cultures elaborated with Malvar must I and must II. This PCA confirmed the evidence given by the analytical assays, making it possible to confirm a higher similarity between mixed cultures and their respective controls in contrast with the greater differences found in sequential cultures (**Figure 3**).

### Sensory Profile of the Produced Wines

**FIGURE 4:**
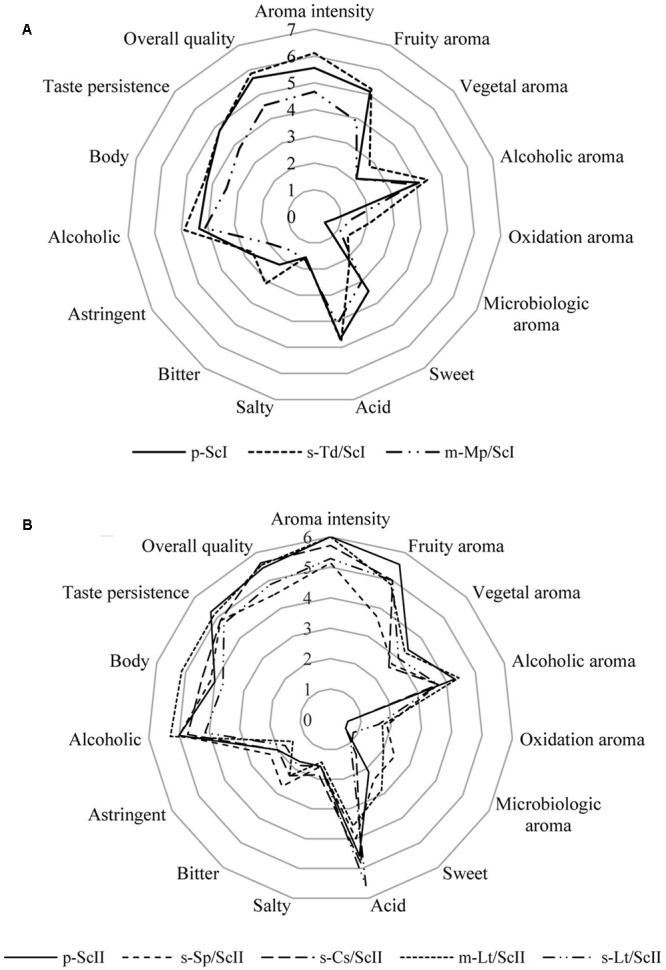
Cobweb diagrams of mean sensory scores of wines made with *Saccharomyces* and non-*Saccharomyces* combinations. **(A)** Cobweb graph of wines: p-ScI, s-Td/ScI, and m-Mp/ScI; and **(B)** cobweb graph of wines: p-ScII, s-Sp/ScII, s-Cs/ScII, m-Lt/ScII, and s-Lt/ScII. Abbreviations related with the type of culture employed and the yeast strains are explained in **Figure 2**.

Wines elaborated were tested by skilled judges from the IMIDRA Institute as the sensorial panel. For fermentations conducted with must I, all panelists were able to distinguish sequential culture of *T. delbrueckii* from the control with a 0.1% significance level by triangle tests. In the case of mixed culture of *M. pulcherrima*/*S. cerevisiae*, tasters differentiated this type of inoculation with respect to the control with a 5% significance level (data not shown). Most panelists considered the sequential culture of *T. delbrueckii* as the best one wine due to its higher aroma intensity, overall quality, and its fruity and floral aroma; also, they denoted its bitter taste (**Figure 4A**). The mixed culture of *M. pulcherrima*/*S. cerevisiae* was described by tasters for its acid and alcoholic character (**Figure 4A**), but also residual sugars in this fermentation (Supplementary Table S1) were detected. The aroma was described by tasters as ripe fruit and banana but, in general, this wine was described as not intense and its lower concentration of volatile compounds compared to the rest of wines can also be seen (Supplementary Table S2).

Furthermore, on fermentations with must II, tasters were able to differentiate the sequential culture of *S. pombe*/*S. cerevisiae* (s-Sp/ScII), the sequential culture of *C. stellata*/*S. cerevisiae* (s-Cs/ScII), and the mixed culture of *L. thermotolerans*/*S. cerevisiae* (s-Lt/ScII) from the control with a 5% significance level by triangle tests; and, the sequential culture of *L. thermotolerans*/*S. cerevisiae* was differentiated with a 1% significance level through the same tests (data not shown). However, there was no clear preference on sensorial analysis; three of the seven panelists preferred the sequential culture of *C. stellata*/*S. cerevisiae*, and two of them chose the mixed culture of *L. thermotolerans*/*S. cerevisiae*, while the other two preferred the sequential culture of *L. thermotolerans* by descriptive analysis.

Sequential culture of *C. stellata* was described by tasters as a wine with a pleasant fruity (green apple, grapefruit) and floral aroma; it was denoted as fresh and full-bodied on the palate (**Figure 4B**).

*Lachancea thermotolerans* in sequential and mixed cultures were well-accepted by tasters (**Figure 4B**). The mixed culture was noted for an intense flavor, balanced acidity, and alcohol with slight sweetness and full body. Its aroma was described as lemon, apple, and nut notes and high aroma intensity. Instead, the sequential culture of *L. thermotolerans* presented the highest acidity of all wines (**Figure 4B**) due to its higher lactic acid content (Supplementary Table S1). Tasters highlighted its fruity (ripe fruit) and floral aroma and freshness on the palate.

Finally, tasters noted that sequential culture of *S. pombe*/*S. cerevisiae* did not improve the organoleptic characteristics to Malvar wines (**Figure 4B**). This wine was described as acid and bitter, low aromatic intensity with citric notes probably due to ethyl octanoate, and ethyl hexanoate volatile compounds (Supplementary Table S2). Additionally, microbiological aroma was detected by tasters in this culture.

## Discussion

In this study, we quantified the evolution of inoculated non-*Saccharomyces* and *Saccharomyces* populations during alcoholic fermentation in different combinations between strains of different species in a natural must of a white grape Malvar variety. A rapid culture-independent qPCR method for detection and enumeration of different yeasts was applied in Malvar wine fermentations. Four pairs of primers were designed in this work into the variable D1/D2 domains of the 26S ribosomal DNA gene to the strains *S. pombe* CLI 1079, *C. stellata* CLI 920, *M. pulcherrima* CLI 457, and *L. thermotolerans* 9-6C; this region has previously been used to develop qPCR methods for several yeasts (Andorrà et al., 2010; Albertin et al., 2014). Two other pair of primers were designed by Zott et al. (2010) to the ITS region of rDNA, and this region is widely used in yeast species identification due to the high degree of interspecies sequence variations (Esteve-Zarzoso et al., 1999; Schoch et al., 2012). These qPCR species-specific primers showed an excellent specificity with all wine yeasts tested and did not amplify other representative wine species. Moreover, standard curves elaborated with the different yeast strains presented high efficiencies, and good detection limits, we enumerated the concentration of 10^3^ cells mL^-1^, and the trials were linear over five orders of magnitude.

The *T. delbrueckii* CLI 918 strain has been utilized to study the matrix influence in the efficiency of qPCR system. Our results were able to show that the matrix of red wine influences on the PCR amplification or on the DNA extraction and purification, due presumably to its much higher proportion of polyphenols. It is known that wine is a complex matrix that presents various PCR inhibitors (Zoecklein et al., 1999; Phister and Mills, 2003), such as major compounds as polyphenols, tannins, and polysaccharides. The efficiency obtained on qPCR analysis from red wine is lower than from white wine and YPD medium, although these values are similar in all cases and without statistical significance. Some authors have reported problems of amplification with DNA isolated directly from wine (Phister and Mills, 2003; Martorell et al., 2005). The assay performed here helped to check that the wine matrix did not significantly influence in the efficiency of the qPCR analysis. According to our results the construction of standard curves in different matrices do not substantially modify the results, and any matrix can be used to quantify the yeast populations from wine fermentation.

It had long been considered that the non-*Saccharomyces* yeasts are present at the beginning of alcoholic fermentation, being replaced by *S. cerevisiae* which has a high capacity to take over the process. In this work, the dynamics of five non-*Saccharomyces* yeasts in co-inoculation with *S. cerevisiae* have been analyzed, revealing that these non-*Saccharomyces* species were present throughout the fermentation process. If they are present during fermentation we expected contribution to the chemical and sensory attributes of the final wines. However, even though these five non-*Saccharomyces* strains were present during fermentation, *S. cerevisiae* was the most abundant yeast under any of the co-cultures tested at the end of the fermentations. Different mechanisms have been described to explain the dominance of *S. cerevisiae* over other competitors during wine fermentation, i.e., cell-to-cell contact (Nissen et al., 2003); competition for nutrients (Taillandier et al., 2014; Kemsawasd et al., 2015b; Lleixà et al., 2016); secretion of toxic compounds (Pérez-Nevado et al., 2006; Branco et al., 2015; Ramírez et al., 2015; Wang et al., 2016), or changes in the medium (Goddard, 2008; Salvadó et al., 2011). These effects caused by *S. cerevisiae* metabolite production and changes in the medium could provide an explanation for the decrease of *M. pulcherrima* and *C. stellata* and the increase and persistence of *T. delbrueckii, S. pombe*, and *L. thermotolerans* belong to the fermentation, due to their higher fermentative power (García et al., 2017) in relation to the amount of alcohol produced by the yeast species (Lopes et al., 2006) and, therefore, related to their alcohol tolerance (Ciani et al., 2016). In the case of *L. thermotolerans*, the enhancement of total acidity produced by this species can also influence in the growth of *S. cerevisiae* and other yeast species. However, the sensibility to these toxic compounds has been described as species- and strain-specific (Wang et al., 2016).

The multi-starter fermentations, combining both non-*Saccharomyces* yeasts and *S. cerevisiae* species able to complete the fermentation, are being studied in depth. All these yeast interaction studies have been increased to explain yeast–yeast interactions and their underlying mechanisms in the increasing use of controlled mixed cultures (Ciani et al., 2010; Ciani and Comitini, 2015). These studies have also been driven by the presence of viable and non-culturable microorganisms in wine samples (Millet and Lonvaud-Funel, 2000; Divol and Lonvaud-Funel, 2005), and may have a false idea about the number of non-*Saccharomyces* species on microbiological methods based on plating (Serpaggi et al., 2012; Wang et al., 2016). In this study, the counts obtained by qPCR were contrasted with plating in YPD non-selective medium and LYS medium (data not shown), a selective medium for non-*Saccharomyces* yeasts. Generally, the yeast populations observed in LYS agar were higher than those obtained by qPCR. This greater growth on LYS medium, could be explained by the growth of other non-*Saccharomyces* yeasts present in the non-sterile Malvar must. This fact is in agreement with the results obtained by Phister and Mills (2003) in a *Dekkera bruxellensis* study.

Differences on the evolution of *Saccharomyces* and non-*Saccharomyces* yeasts have been observed depending on the type of inoculation. In the mixed culture of *M. pulcherrima*/*S. cerevisiae*, the *M. pulcherrima* CLI 457 population started to decrease at 24 h in contrast with the increase of *S. cerevisiae* counts studied by qPCR. The antagonist effect of *M. pulcherrima* on several yeasts, including *S. cerevisiae*, which leads to delays in the fermentation, has been studied (Nguyen and Panon, 1998; Türkel and Ener, 2009). This phenomenon was due to a killer effect linked to pulcherrimin pigment produced by *M. pulcherrima* strains, Türkel and Ener (2009) found three strains of *M. pulcherrima* (UMY12, UMY14, and UMY15) that produce the same amount of the pigment pulcherrimin, but their antimicrobial activities showed important variations. Different distinct biotypes within the *M. pulcherrima* species with respect the pulcherrimin production were identified by Pallmann et al. (2001). However, it has recently been described a difficulty in classifying *Metschnikowia fructicola* species since this species is not distinguishable from *Metschnikowia andauensis* and other species of the *M. pulcherrima* clade because of a possible heterogeneity of rRNA repeats (Cordero-Bueso et al., 2017). For this reason, we keep the original designation for this yeast strain, keeping the same yeast species name described on the published document by Arroyo et al. (2010). However, the variable D1/D2 domain of this strain was sequenced by Macrogen to be identified with 99% of sequence identity as *M. pulcherrima* and its sequence included in GenBank Database (accession number MF001378). Our results showed that the mixed culture of *M. pulcherrima*/*S. cerevisiae* finished with a high level of reducing sugars, in the same way that happened in co-cultures with these strains in laboratory scale fermentations (García et al., 2017), so it could be possible that the *M. pulcherrima* CLI 457 strain had a negative effect on the fermentative capacity of the *S. cerevisiae* CLI 889 strain. Instead, sequential fermentation with *T. delbrueckii* and *S. cerevisiae* finished with sugar values lower than 4 g L^-1^ as at laboratory level (García et al., 2017). Therefore, the fermentative capacity of *T. delbrueckii* in the first days seems to influence in the low sugar content of final wines, independently of the scale of fermentations, which is in agreement with results obtained by Puertas et al. (2016).

Some authors have reported the competition mechanisms between *L. thermotolerans* and *S. cerevisiae* in mixed culture. Hansen et al. (2001) found that oxygen increases the competitiveness between *L. thermotolerans* CBS 2803 and *S. cerevisiae* Saint Georges S101 strains in mixed culture. In the same way, Nissen et al. (2004) concluded that *S. cerevisiae* Saint Georges S101 is able to grow and ferment more efficiently under oxygen-limited conditions present during wine fermentation in comparison with *L. thermotolerans* CBS 2803 and *T. delbrueckii* CBS 3085. Although other previous studies (Nissen and Arneborg, 2003; Nissen et al., 2003) showed that the death of *L. thermotolerans* in mixed culture with *S. cerevisiae* was induced by a cell-to-cell contact mediated mechanism with the same strains used by Nissen et al. (2004). Finally, Kemsawasd et al. (2015a) concluded that cell-to-cell contact and antimicrobial peptides play a combined role in the death of *L. thermotolerans* CBS 2803 in mixed fermentation with *S. cerevisiae* Saint Georges S101 strain. Our strain of *L. thermotolerans* in mixed culture showed a loss of viability most pronounced, although both populations decreased during fermentation process from day 3.

In sequential cultures, the *S. cerevisiae* population found in Malvar wine in the first 24 h of fermentation were low, between 10^2^ and 10^3^ cells mL^-1^. It can be seen that native *Saccharomyces* yeasts of the cellar environmental started to grow on the following days, but when *S. cerevisiae* CLI 889 was inoculated (day 13), this strain causes a progressive fall in the density until the end of sequential fermentations. It is well-known that *S. cerevisiae* yeasts are very competitive and normally dominates the fermentation due to its fast growth, efficient glucose competition, good ability to produce ethanol, and a higher tolerance to environmental stresses (Piškur et al., 2006). In this study, the growth of *S. cerevisiae* CLI 889 after its inoculation may have been affected by environmental factors, such as a low controlled temperature (18°C) during the fermentation process, a different availability of nutrients in the musts, and a wine elaboration without the addition of nutrients. After microsatellites multiplex analysis, the presence of the inoculated *S. cerevisiae* CLI 889 strain at the end of fermentation together with other *S. cerevisiae* strains could be confirmed; although in sequential culture, *S. cerevisiae* CLI 889 was found in lower percentage than in mixed cultures at the end of fermentation.

Nutrient content of the musts can modulate the yeast populations, the time of fermentation and secondary metabolites produced during alcoholic fermentation (Beltran et al., 2005; Andorrà et al., 2012; Kemsawasd et al., 2015b). In grape must, nitrogen is considered the main limiting nutrient for optimized growth and good fermentation performance (Bisson, 1999). We could observe when Malvar must II was used in the elaboration of wines, the fermentation length was increased in the cultures (40 days) compared to the elaborations with must I that finished in 32 days; the higher YAN content of must I (218 mgN L^-1^) than must II (100 mgN L^-1^) could have influence in the fermentation rate in agreement with other studies (Bely et al., 1990; Monteiro and Bisson, 1992; Beltran et al., 2005). Medina et al. (2012) noticed a negative effect of non-*Saccharomyces* yeasts on nutrient availability for *S. cerevisiae* reducing its ability to grow, especially when it was sequentially inoculated. In the tested sequential fermentations, it could be possible that the YAN consumption by non-*Saccharomyces* would explain the slow growth of *S. cerevisiae* CLI 889, although *S. cerevisiae* population was eventually greater at final of fermentation in all cases, since it is well-known that *S. cerevisiae* strains show a favorable adaptation to the nitrogen-limited wine fermentation environment (Marsit et al., 2015). Additionally, a higher alcohols production (isobutanol, isoamyl alcohol, metionol, and β-phenylethyl alcohol) has been noted in fermentations elaborated with Malvar must II. This is related to the nitrogen concentration, the less nitrogen there is available in the fermentation medium, the more higher alcohols are produced (Beltran et al., 2005; Andorrà et al., 2012). The higher alcohols, along with glycerol, are the end-products of reductive pathway alternatives to the ethanol products. However, we did not detect in all co-cultures a significant decrease in the ethanol content with regard to their controls. Other volatile compounds as acetates, ethyl esters, and 1-propanol have also presented positive correlation with the level of nitrogen in the fermentation process (Rapp and Versini, 1995), this correlation can be observed for most of these compounds when the wines were elaborated with must I.

In terms of glycerol content, we can confirm the use of the tested non-*Saccharomyces* strains provides an enhancement of glycerol both at laboratory scale and at the pilot scale with the exception of *L. thermotolerans* 9-6C that did not produce high concentrations with respect to its controls at both scales. It is well-known that several non-*Saccharomyces* yeasts can considerably increase the glycerol concentrations in wine (Soden et al., 2000; Cominiti et al., 2011; Englezos et al., 2015; Benito et al., 2016b). Glycerol is one of the major compounds produced during wine fermentation, and it is important in yeast metabolism for regulating the redox potential in the cell (Prior et al., 2000). This compound contributes to mouth-feel, sweetness, and complexity in wines (Ciani and Maccarelli, 1998), but its production is usually linked to increased acetic acid production (Prior et al., 2000). In our results, the volatile acidity values measured as grams per liter of acetic acid, were kept low, especially at the pilot scale, with a particular decline in volatile acidity produced by *T. delbrueckii* CLI 918 in sequential culture.

In respect of the oenological parameters studied, the behavior of the yeast strains and the wine styles were similar regardless of the scale of fermentation tested. However, due to the type of vinification being different, some parameters changed at the pilot scale. Most of the wines can be considered as dry since their sugar content was less than 4 g L^-1^ at final of fermentation (Belitz and Grosch, 1999), with the exception of pure culture of *S. cerevisiae* p-ScI and mixed culture of *M. pulcherrima*/*S. cerevisiae* (m-Mp/ScI) (Supplementary Table S1). Generally, volatile acidity values are lower for all co-cultures in this work.

Sequential culture of *T. delbrueckii*/*S. cerevisiae* (s-Td/ScI), in comparison with its control (p-ScI), was distinguished for a significant decrease in volatile acidity (0.34 g L^-1^) and an increase of glycerol content (Supplementary Table S1). In relation with aromatic compounds, sequential culture of *T. delbrueckii* presented higher concentration of β-phenylethyl alcohol, and esters, such as ethyl butyrate, ethyl isovalerate, isoamyl acetate, ethyl hexanoate, and 2-phenylethyl acetate (Supplementary Table S2) associated with the fruity and floral character of this wine.

In relation with cultures elaborated with *L. thermotolerans* 9-6C and *S. cerevisiae* CLI 889, the effect of *L. thermotolerans* on oenological and sensorial properties of wines (increase of lactic acid, glycerol, and β-phenylethyl alcohol) depends on the way of inoculation with *S. cerevisiae* (Kapsopoulou et al., 2007; Gobbi et al., 2013). We observed a higher lactic acid and β-phenylethyl alcohol content in sequential culture due to *L. thermotolerans* 9-6C growth before *S. cerevisiae* CLI 889 inoculation. *L. thermotolerans* seems to be dominant over *S. cerevisiae* due to the significant enhancement in total acidity and, consequently, a decrease of pH. In contrast, this behavior appears to be softened in mixed culture. This result contrasts with other studies (Gobbi et al., 2013; Benito et al., 2016a) that also observed this pattern of competitiveness in the different inoculation strategies with *L. thermotolerans* and *S. cerevisiae*.

Our results showed that *C. stellata* CLI 920, along with *L. thermotolerans* 9-6C, are strains that produce lactic acid and, therefore, they increase the total acidity, both at the laboratory scale using sterile Malvar must and at the pilot scale. This production could be related with the higher concentration of ethyl lactate observed in both sequential inoculations since this ester is produced by esterification from acid lactic and ethanol (Inaba et al., 2009; Delgado et al., 2010). Higher concentrations of ethyl lactate after the use of co-cultures with *L. thermotolerans* and *S. cerevisiae* have been documented by other authors (Cominiti et al., 2011; Gobbi et al., 2013; Benito et al., 2015, 2016a).

In relation to the *S. pombe* strain, we tested in this work the *S. pombe* CLI 1079 yeast strain instead of the CLI 1085 strain used at laboratory scale due to the low growth capacity of this latest strain, that did impossible a successful *pied de cuve* at the pilot scale. The *S. pombe* CLI 1079 in sequential culture were able to finish the fermentation with residual sugars less than 4 g L^-1^; this strain presented a low consumption of the malic acid at the pilot scale, ending the fermentation with 1.00 g L^-1^ of malic acid, a value slightly lower than its control (p-ScII). Additionally, glycerol content was higher than the control. This culture presented an elevated concentration of β-phenylethyl alcohol and the highest values of alcohols. Volatile compounds associated with cheese and butter aromas were higher in sequential culture of *S. pombe* than the control p-ScII.

## Conclusion

We can confirm that the inoculation strategies conducted at the laboratory scale produce a notable improvement in the quality of regional Malvar wines at the pilot scale also. Tasters were able to distinguish the different elaborations with respect the controls and most appreciated wines by tasting panel were those elaborated in sequential cultures with *T. delbrueckii* CLI 918/*S. cerevisiae* CLI 889 and *C. stellata* CLI 920/*S. cerevisiae* CLI 889 and, mixed and sequential cultures with *L. thermotolerans* 9-6C in combination with the *S. cerevisiae* CLI 889 strain. Sequential cultures have produced more different wines with respect to the controls, providing organoleptic properties associated with the non-*Saccharomyces* strains, but more studies need to be carried out varying the moment of inoculation of *S. cerevisiae* strain in these cultures to prevent native *S. cerevisiae* growth on musts, and the reduction of the fermentation time. This work provides the basis for the implementation of new biotechnological strategies for improving Malvar wine quality and it can be tested in commercial wineries.

## Author Contributions

MG, TA, and BE-Z designed the experiments, analyzed the results, discussion of the results and wrote the manuscript. MG, JC, and JMC performed experiments and analyzed results.

## Conflict of Interest Statement

The authors declare that the research was conducted in the absence of any commercial or financial relationships that could be construed as a potential conflict of interest.

## Footnotes

1 http://www.ncbi.nlm.nih.gov

2 http://www.primer3plus.com

3 https://www-s.nist.gov/dnaAnalysis/primerToolsPage.do
