# Supplementary material for: Yeast Monitoring of Wine Mixed or Sequential Fermentations Made by Native Strains from D.O. “Vinos de Madrid” Using Real-Time Quantitative PCR
Source: Front Microbiol. 2017 Dec 20;8:2520. doi: 10.3389/fmicb.2017.02520 (PMC5742323; doi:10.3389/fmicb.2017.02520)
Supplement: Supplementary file 1 [file Table_1.docx]

Supplementary Material

Yeast Monitoring of Wine Mixed or Sequential Fermentations Made by Native Strains from D.O. “Vinos de Madrid” Using Real Time Quantitative PCR

Margarita García^1^, Braulio Esteve-Zarzoso^2*^, Julia Crespo^1^, Juan Mariano Cabellos^1^ and Teresa Arroyo^1*^

*** Correspondence:**  Braulio Esteve-Zarzoso: braulio.esteve@urv.cat

Teresa Arroyo: teresa.arroyo@madrid.org

Table S1 (Supplementary Material). Principal oenological parameters at the end of the different fermentations. Values are means ± standard deviations of triplicate fermentations. * Means statistically different from the control, p < 0.05. ^a^Abbreviations related with the type of culture employed and the yeast strains are explained in Figure 2. ^b^Fermentations with *S. cerevisiae* pure cultures (p-ScI and p-ScII) were taken as the control in each must batch (must I and must II).

|  |  | |  |  |  |  |  | |  |  |
| --- | --- | --- | --- | --- | --- | --- | --- | --- | --- | --- |
| **Type of culture^a^** | | **Ethanol** | **pH** | **Volatile acidity** | **Total acidity** | **Reducing sugars** | | **Glycerol** | **Malic acid** | **Lactic acid** |
|  | %, *v*/*v* | |  | g/L acetic acid | g/L tartaric acid | g/L | g/L | | g/L | g/L |
|  |  | |  |  |  |  |  | |  |  |
|  |  | |  |  |  |  |  | |  |  |
| p-ScI^b^ | 13.55 ± 0.05 | | 3.03 ± 0.01 | 0.56 ± 0.01 | 6.76 ± 0.01 | 9.86 ± 0.23 | 6.15 ± 0.08 | | 0.73 ± 0.06 | <0.50 |
|  |  | |  |  |  |  |  | |  |  |
| s-Td/ScI | 13.50 ± 0.09 | | 3.04 ± 0.03 | 0.34 ± 0.03* | 6.88 ± 0.22 | 2.95 ± 0.66* | 7.00 ± 0.84 | | 0.47 ± 0.06* | <0.50 |
|  |  | |  |  |  |  |  | |  |  |
| m-Mp/ScI | 13.42 ± 0.09 | | 3.03 ± 0.01 | 0.58 ± 0.04 | 6.76 ± 0.12 | 9.17 ± 0.69 | 6.55 ± 0.08 | | 0.30 ± 0.01* | <0.50 |
| p-ScII^b^ | 13.00 ± 0.00 | | 3.30 ± 0.00 | 0.40 ± 0.00 | 5.30 ± 0.10 | 3.75 ± 0.01 | 4.70 ± 0.12 | | 1.10 ± 0.09 | <0.50 |
|  |  | |  |  |  |  |  | |  |  |
| s-Sp/ScII | 13.00 ± 0.10 | | 3.30 ± 0.00 | 0.53 ± 0.06* | 5.67 ± 0.12* | 3.90 ± 0.52 | 5.37 ± 0.09* | | 1.00 ± 0.35 | <0.50 |
|  |  | |  |  |  |  |  | |  |  |
| s-Cs/ScII | 13.20 ± 0.00* | | 3.30 ± 0.00 | 0.50 ± 0.00* | 6.23 ± 0.12* | 2.37 ± 0.15* | 5.41 ± 0.08* | | 0.60 ± 0.04 | 1.10 ± 0.06* |
|  |  | |  |  |  |  |  | |  |  |
| m-Lt/ScII | 13.30 ± 0.00* | | 3.30 ± 0.00 | 0.50 ± 0.00* | 5.40 ± 0.10 | 3.70 ± 0.10 | 4.86 ± 0.24 | | 1.00 ± 0.01 | <0.50 |
|  |  | |  |  |  |  |  | |  |  |
| s-Lt/ScII | 13.07 ± 0.12 | | 3.10 ± 0.06* | 0.57 ± 0.06* | > 7.00* | 2.67 ± 0.65* | 4.62 ± 0.25 | | <0.20* | 2.40 ± 0.60* |
|  |  | |  |  |  |  |  | |  |  |
