# Supplementary material for: Yeast Monitoring of Wine Mixed or Sequential Fermentations Made by Native Strains from D.O. “Vinos de Madrid” Using Real-Time Quantitative PCR
Source: Front Microbiol. 2017 Dec 20;8:2520. doi: 10.3389/fmicb.2017.02520 (PMC5742323; doi:10.3389/fmicb.2017.02520)
Supplement: Supplementary file 2 [file Table_2.docx]

Supplementary Material

Yeast Monitoring of Wine Mixed or Sequential Fermentations Made by Native Strains from D.O. “Vinos de Madrid” Using Real Time Quantitative PCR

Margarita García^1^, Braulio Esteve-Zarzoso^2*^, Julia Crespo^1^, Juan Mariano Cabellos^1^ and Teresa Arroyo^1*^

*** Correspondence:**  Braulio Esteve-Zarzoso: braulio.esteve@urv.cat

Teresa Arroyo: teresa.arroyo@madrid.org

| **Compound** | **ODE** | **OTV** | **Type of culture^a^** | | | | | | | |
| --- | --- | --- | --- | --- | --- | --- | --- | --- | --- | --- |
|  |  |  | p-ScI^b^ | s-Td/ScI | m-Mp/ScI | p-ScII^b^ | s-Sp/ScII | s-Cs/ScII | m-Lt/ScII | s-Lt/ScII |
| 1-Propanol | Alcohol, ripe fruit | 9 | 5.11 ± 0.43 | 6.16 ± 1.19 | 4.35 ± 0.80 | 1.62 ± 0.40 | 2.02 ± 0.07 | 2.16 ± 0.28 | 1.84 ± 0.22 | 2.01 ± 0.14 |
| 1-Butanol | Soap, fatty, diesel | 150 | 0.81 ± 0.11 | 1.18 ± 0.19* | 0.29 ± 0.07* | 0.49 ± 0.08 | 0.47 ± 0.04 | 0.79 ± 0.21 | 0.55 ± 0.08 | 0.77 ± 0.08 |
| Isobutanol | Bitter, fusel, alcohol | 40 | 17.65 ± 1.62 | 20.91 ± 2.70 | 11.27 ± 0.45* | 29.42 ± 2.28 | 31.62 ± 5.71 | 33.35 ± 4.23 | 27.85 ± 3.19 | 25.91 ± 2.48 |
| Isoamyl alcohol | Harsh, bitter | 30 | 115.83 ± 10.76 | 183.84 ± 17.09* | 72.74 ± 0.80* | 267.85 ± 17.10 | 337.65 ± 8.21* | 298.90 ± 9.02 | 256.12 ± 10.42 | 299.52 ± 13.36 |
| (Z)-3-Hexen-1-ol | Lemon, fresh | 0.4 | 0.13 ± 0.01 | 0.11 ± 0.04 | 0.09 ± 0.02 | 0.08 ± 0.01 | 0.12 ± 0.01 | 0.12 ± 0.04 | 0.09 ± 0.01 | 0.12 ± 0.00 |
| 1-Hexanol | Green grass, fresh | 8 | 1.22 ± 0.06 | 0.92 ± 0.13* | 0.85 ± 0.05* | 1.91 ± 0.06 | 2.50 ± 0.05 | 2.96 ± 0.78 | 2.00 ± 0.41 | 2.75 ± 0.14 |
| Metionol | Garlic | 1 | 0.21 ± 0.03 | 0.57 ± 0.05* | 0.15 ± 0.02 | 0.65 ± 0.08 | 1.87 ± 0.26* | 1.75 ± 0.63 | 0.66 ± 0.21 | 1.55 ± 0.60 |
| Benzyl alcohol | Pleasant, soft | 200 | 0.01 ± 0.00 | 0.13 ± 0.00* | 0.04 ± 0.00 | 0.06 ± 0.02 | 0.06 ± 0.01 | 0.11 ± 0.05 | 0.05 ± 0.01 | 0.06 ± 0.01 |
| β-Phenylethyl alcohol | Flowery, roses | 14 | 8.81 ± 0.56 | 20.41 ± 3.15* | 6.53 ± 0.39 | 15.50 ± 1.90 | 55.74 ± 3.26* | 56.14 ± 9.00* | 32.75 ± 8.62 | 69.21 ± 8.72* |
| Σ Alcohols |  |  | 149.78 ± 13.47 | 234.22 ± 23.64* | 96.31 ± 0.61* | 317.58 ± 21.92 | 432.05 ± 17.59* | 396.51 ± 19.22* | 321.91 ± 28.27 | 401.90 ± 6.96* |
| Ethyl butyrate | Fruity, sweet, apple | 0.02 | 0.41 ± 0.04 | 0.63 ± 0.07* | 0.33 ± 0.00 | 0.42 ± 0.02 | 0.34 ± 0.07 | 0.39 ± 0.08 | 0.44 ± 0.09 | 0.29 ± 0.02 |
| Ethyl isovalerate | Fruity, sweet, banana | 0.003 | 0.15 ± 0.01 | 0.36 ± 0.03* | 0.11 ± 0.02 | 0.12 ± 0.01 | 0.18 ± 0.03 | 0.24 ± 0.07 | 0.14 ± 0.03 | 0.24 ± 0.07 |
| Ethyl isobutyrate | Fruity, pineapple | 0.015 | 0.00 ± 0.00 | 0.00 ± 0.00 | 0.00 ± 0.00 | 0.00 ± 0.00 | 0.00 ± 0.00 | 0.14 ± 0.02* | 0.01 ± 0.00 | 0.05 ± 0.01 |
| Isoamyl acetate | Banana, sweet, fruity | 0.03 | 2.04 ± 0.44 | 5.75 ± 0.98* | 1.20 ± 0.04 | 1.59 ± 0.06 | 1.07 ± 0.21 | 1.41 ± 0.20 | 1.58 ± 0.33 | 1.21 ± 0.15 |
| Ethyl hexanoate | Pineapple, apple | 0.014 | 0.64 ± 0.16 | 0.81 ± 0.05 | 0.45 ± 0.02 | 0.73 ± 0.02 | 0.91 ± 0.13 | 1.05 ± 0.30 | 0.69 ± 0.14 | 0.85 ± 0.05 |
| Ethyl-3-hydroxybutyrate | Fruity | 20 | 0.11 ± 0.02 | 0.29 ± 0.05* | 0.09 ± 0.00 | 0.12 ± 0.02 | 0.14 ± 0.00 | 0.28 ± 0.08* | 0.13 ± 0.02 | 0.22 ± 0.05 |
| Hexyl acetate | Fruity, green, pear | 1 | 0.16 ± 0.03 | 0.18 ± 0.01 | 0.08 ± 0.01* | 0.14 ± 0.00 | 0.11 ± 0.03 | 0.10 ± 0.02 | 0.10 ± 0.01 | 0.08 ± 0.01* |
| 2-Phenylethyl acetate | Flowery, lilac | 0.25 | 0.07 ± 0.02 | 4.09 ± 0.27* | 2.57 ± 0.18* | 0.17 ± 0.01 | 0.22 ± 0.03 | 0.19 ± 0.08 | 0.10 ± 0.02 | 0.16 ± 0.01 |
| Diethyl succinate | Camphor | 100 | 0.04 ± 0.01 | 0.01 ± 0.00 | 0.04 ± 0.00 | 0.06 ± 0.01 | 0.14 ± 0.02* | 0.04 ± 0.01 | 0.06 ± 0.02 | 0.08 ± 0.02 |
| Ethyl octanoate | Fresh, flowery, pineapple | 0.58 | 0.50 ± 0.12 | 0.46 ± 0.01 | 0.42 ± 0.00 | 0.62 ± 0.14 | 0.88 ± 0.10 | 1.03 ± 0.28 | 0.65 ± 0.20 | 0.62 ± 0.13 |
| Ethyl lactate | Lactic | 154 | 2.07 ± 0.14 | 3.09 ± 0.32* | 1.60 ± 0.05 | 2.89 ± 0.18 | 3.07 ± 0.08 | 35.54 ± 0.02* | 14.24 ± 0.21 | 110.71 ± 6.48* |
| Σ Esters |  |  | 6.18 ± 0.90 | 15.67 ± 1.42* | 6.88 ± 0.20 | 6.86 ± 0.16 | 7.08 ± 0.46 | 40.40 ± 1.26* | 18.15 ± 2.36 | 114.50 ± 6.69* |
| Isobutyric acid | Rancid, butter, cheese | 0.05 | 0.53 ± 0.05 | 0.94 ± 0.15* | 0.34 ± 0.03 | 1.46 ± 0.22 | 2.50 ± 0.68 | 4.20 ± 0.87* | 1.39 ± 0.30 | 2.64 ± 0.60 |
| Butyric acid | Butter, cheese, stinky | 0.173 | 0.16 ± 0.02 | 0.15 ± 0.00 | 0.14 ± 0.01 | 0.14 ± 0.01 | 0.18 ± 0.00 | 0.19 ± 0.03* | 0.14 ± 0.02 | 0.16 ± 0.02 |
| Isovaleric acid | Cheese | 0.033 | 0.04 ± 0.00 | 0.06 ± 0.01 | 0.15 ± 0.10 | 1.52 ± 0.16 | 3.39 ± 0.17* | 3.96 ± 1.01* | 1.37 ± 0.09 | 3.27 ± 0.72* |
| Hexanoic acid | Cheese | 0.42 | 0.02 ± 0.00 | 0.05 ± 0.02 | 0.02 ± 0.01 | 3.78 ± 0.45 | 5.22 ± 0.31 | 5.89 ± 1.98 | 4.08 ± 0.96 | 4.06 ± 0.41 |
| Octanoic acid | Sweet, cheesy | 0.5 | 0.00 ± 0.00 | 0.00 ± 0.00 | 0.00 ± 0.00 | 7.62 ± 1.11 | 9.59 ± 1.67 | 11.29 ± 3.82 | 8.06 ± 1.59 | 7.54 ± 1.10 |
| Decanoic acid | Rancid, fatty | 1 | 0.78 ± 0.15 | 1.04 ± 0.02* | 0.57 ± 0.02 | 1.23 ± 0.20 | 1.50 ± 0.20 | 2.41 ± 0.66* | 1.18 ± 0.38 | 1.97 ± 0.35 |
| Σ Acids |  |  | 1.50 ± 0.21 | 2.21 ± 0.17* | 1.20 ± 0.08 | 15.76 ± 1.62 | 22.38 ± 1.79 | 27.94 ± 8.83* | 16.23 ± 3.46 | 19.65 ± 1.43 |
| Diacetyle | Butter | 0.1 | 0.09 ± 0.01 | 0.11 ± 0.03 | 0.05 ± 0.00 | 0.44 ± 0.04 | 0.49 ± 0.13 | 0.52 ± 0.16 | 0.42 ± 0.04 | 0.38 ± 0.13 |
| Furfural | Bread, toasty, candy | 15 | 0.18 ± 0.03 | 0.16 ± 0.05 | 0.11 ± 0.02 | 0.10 ± 0.03 | 0.01 ± 0.00 | 0.02 ± 0.00 | 0.08 ± 0.02 | 0.05 ± 0.03 |
| Benzaldehyde | Sweet, candy, wood | 5 | 0.07 ± 0.01 | 0.07 ± 0.01 | 0.05 ± 0.00 | 0.00 ± 0.00 | 0.00 ± 0.00 | 0.00 ± 0.00 | 0.00 ± 0.00 | 0.00 ± 0.00 |
| Phenylacetaldehyde | Roses | 1 | 0.16 ± 0.01 | 0.07 ± 0.02 | 0.03 ± 0.01 | 0.00 ± 0.00 | 0.00 ± 0.00 | 0.00 ± 0.00 | 0.00 ± 0.00 | 0.00 ± 0.00 |
| Acetoine | Butter | 150 | 2.24 ± 0.25 | 0.09 ± 0.04* | 0.18 ± 0.08* | 1.87 ± 0.70 | 2.12 ± 0.25 | 2.46 ± 0.52 | 1.51 ± 0.43 | 2.14 ± 0.28 |
| Σ Aldehydes/Ketones |  |  | 2.73 ± 1.06 | 0.49 ± 0.17* | 0.42 ± 0.06* | 2.41 ± 0.77 | 2.61 ± 0.33 | 3.00 ± 1.10 | 2.01 ± 0.82 | 2.57 ± 0.63 |
| γ-Butyrolactone | Coconut | 35 | 2.07 ± 0.30 | 2.06 ± 0.28 | 1.26 ± 0.01* | 4.23 ± 0.72 | 4.58 ± 0.73 | 5.10 ± 1.02 | 5.04 ± 1.33 | 3.30 ± 0.87 |

Table S2 (Supplementary Material). Volatile compounds (mg L^-1^), ODE (odour description), and OTV (odour threshold value, mg L^-1^, Balboa-Lagunero *et al*. 2013) of different types of inoculation with *Saccharomyces* and non-*Saccharomyces* strains (Td, *T. delbrueckii*; Mp, *M. pulcherrima*; Sp, *S. pombe*; Cs, *C. stellata*; and Lt, *L. thermotolerans*). Values are the mean ± SD of triplicate fermentations. * Means statistically different from the control, p < 0.05. ^a^Abbreviations related with the type of culture employed and the yeast strains are explained in Figure 2. ^b^Fermentations with *S. cerevisiae* pure cultures (p-ScI and p-ScII) were taken as the control in each must batch (must I and must II).
